# Prognostic Values of microRNAs in Colorectal Cancer

**Published:** 2007-02-07

**Authors:** Yaguang Xi, Andrea Formentini, Minchen Chien, David B. Weir, James J. Russo, Jingyue Ju, Marko Kornmann, Jingfang Ju

**Affiliations:** 1 The Mitchell Cancer Institute, University of South Alabama, Mobile, AL 36688; 2 Department of Visceral and Transplantation Surgery, University of Ulm, Steinhoevelstrasse 9, 89075 Ulm, Germany; 3 Columbia Genome Center, Columbia University, New York, NY, 10032

**Keywords:** hsa-miR-200c, micro-RNA, prognosis, colorectal cancer

## Abstract

The functions of non-coding microRNAs (miRNAs) in tumorigenesis are just beginning to emerge. Previous studies from our laboratory have identified a number of miRNAs that were deregulated in colon cancer cell lines due to the deletion of the p53 tumor suppressor gene. In this study, the *in vivo* significance of some of these miRNAs was further evaluated using colorectal clinical samples. Ten miRNAs (*hsa-let-7b, hsa-let-7g, hsa-miR-15b, hsa-miR-181b, hsa-miR-191, hsa-miR-200c, hsa-miR-26a, hsa-miR-27a, hsa-miR-30a-5p and hsa-miR-30c*) were evaluated for their potential prognostic value in colorectal cancer patients. Forty eight snap frozen clinical colorectal samples (24 colorectal cancer and 24 paired normal patient samples) with detailed clinical follow-up information were selected. The expression levels of 10 miRNAs were quantified via qRT-PCR analysis. The statistical significance of these markers for disease prognosis was evaluated using a two tailed paired Wilcoxon test. A Kaplan-Meier survival curve was generated followed by performing a Logrank test. Among the ten miRNAs, *hsa-miR-15b* (p = 0.0278), *hsa-miR-181b* (p = 0.0002), *hsa-miR-191* (p = 0.0264) and *hsa-miR-200c* (p = 0.0017) were significantly over-expressed in tumors compared to normal colorectal samples. Kaplan-Meier survival analysis indicated that *hsa-miR-200c* was significantly associated with patient survival (p = 0.0122). The patients (n = 15) with higher *hsa-miR-200c* expression had a shorter survival time (median survival = 26 months) compared to patients (n = 9) with lower expression (median survival = 38 months). Sequencing analysis revealed that *hsa-miR-181b* (p = 0.0098) and *hsa-miR-200c* (p = 0.0322) expression were strongly associated with the mutation status of the p53 tumor suppressor gene. Some of these miRNAs may function as oncogenes due to their over-expression in tumors. *hsa-miR-200c* may be a potential novel prognostic factor in colorectal cancer.

## Introduction

microRNAs (miRNAs) are non-coding, single-stranded RNAs of ~22 nucleotides, derived from miRNA duplex complexes processed from larger pre-miRNAs by the RNase III enzyme Dicer (Bartel, 2004). One strand of this duplex can associate with the RNA-induced silencing complex (RISC), while the other strand is generally degraded by cellular nucleases. The miRNA-RISC complex has been shown to bind to specific mRNA targets and cause the translational repression or cleavage of these mRNA sequences. Currently, there are several hundred miRNAs that have been identified (Esquela-Kerscher and Slack, 2006). Although miRNA-mediated mRNA degradation occurs in mammals, most miRNAs are thought to use a second mechanism of gene regulation via imperfect base-pairing to the 3′-untranslated regions (3′-UTRs) of their mRNA targets. This results in the repression of target gene expression post-transcriptionally, likely at the translational level of gene expression (Lee et al. 1993; Wienholds and Plasterk, 2005). Recent studies from our laboratory as well as those of other groups have shown that the expression of miRNAs is important in colorectal and other types of cancer (Benard and Douc-Rasy, 2005; Bottoni et al. 2005; Calin et al. 2002; Calin et al. 2005; Cimmino et al. 2005; Cummins et al. 2006; Xi et al. 2006). miRNA regulates gene expression at the post-transcriptional level by perfect or imperfect base pairing with many target mRNA transcripts (Chen and Meister, 2005; Mattick and Makunin, 2005). This provides cells with acute and energy efficient controls to mediate gene expression in response to cellular stresses such as drug treatment. Translation control plays a key role in the mechanism of cellular resistance to anti-cancer drug treatment (Chu et al. 1999; Chu et al. 1991; Fu et al. 1996; Ju et al. 2003; Ju et al. 1999; Mosner et al. 1995). Therefore, it is crucial to understand the roles and regulatory mechanisms of translational control in chemoresistance, and in particular, the roles of miRNAs as translational regulators.

At present, over 300 mammalian miRNAs have been identified and some of them have been shown to play important roles in the regulation of cell differentiation, proliferation and apoptosis. Each individual miRNA can potential regulate mRNA translation for up to several hundred genes via imperfect base pairing based on bioinformatic analysis (Krek et al. 2005). We reasoned that miRNAs may be a better class of biomarkers because of their broad regulatory functions and the ability to measure their expression levels with far better accuracy than is currently achievable for the mRNAs themselves (Draghici et al. 2006).

Studies from our group have demonstrated that p53, in addition to regulating gene expression as a transcription factor, may exert its tumor suppressor gene function by mediating miRNA expression in a colon cancer model (Xi et al. 2006). Over 46% of miRNA promoters contain putative p53 consensus binding site(s). A number of miRNAs were discovered to be deregulated due to the loss of the p53 tumor suppressor in colon cancer cell lines. Some of these miRNAs mediate key mRNA targets such as *Ras, E2F5, c-myc, eIF5A, and PP2B.* These genes have been shown to be key factors in influencing cell cycle control and chemosensitivity (Banerjee et al. 1998; Liu et al. 1989; Sinha et al. 1995). A recent study examined miRNA expression in 16 colon cancer cell lines and 12-matched-pair tumor and non-tumor tissues using the real time qRT-PCR approach (Bandres et al. 2006). In this report, among 156 miRNAs analyzed, six were found to be significantly altered and miR-31 was correlated with tumor staging.

Instead of random screening, we decided to use a rationalized approach based on our expression profiling analysis of HCT-116 (wt-p53) and HCT-116 (null-p53) colon cancer cell lines (Xi et al. 2006) to further evaluate the *in vivo* significance of deregulated miRNAs in relation to chemosensitivity. In the current study, the expression levels of ten miRNAs (*hsa-miR-30a-5p, hsa-miR-181b, hsa-let-7g, hsa-miR-26a, hsa-let-7b, has-miR-15b, has-miR-27a, has-miR-200c, has-miR-191,* and *has-miR-30c*) were investigated to evaluate their clinical relevance in colorectal cancer. 24 normal and paired colorectal cancer specimens were selected as a model for this investigation. These ten miRNAs were chosen based on their high expression levels in tumors displaying p53 deletion, their relationship with p53 and their predicted target mRNAs (e.g. *cytochrome C, ECIP-1, MAPPKKK1, TEM6, E2F5, GATA6, PP2B,* and *eIF5A*). Based on our results, some of these miRNAs may function as oncogenes due to their overexpression in tumors. *hsa-miR-200c* is a potential novel prognostic factor for survival of patients with colorectal cancer. Overexpression of these miRNAs may be due to the loss of p53 tumor suppressor function in tumors.

## Patients and Methods

### Patients and Samples

A total of 48 snap frozen colorectal patient biopsy specimens were selected (24 paired normal and tumor specimens). These patients had undergone surgical resection of primary colorectal adenocarcinoma at the Department of General Surgery, University of Ulm, Germany. Patient consent forms were obtained from every patient according to the institutional regulations. The characteristics of these patients are shown in Table 1. Some were treated with adjuvant 5-FU based chemotherapy and others were treated with palliative high-dose 5-FU/FA.

### RNA Isolation and cDNA synthesis

Total RNAs were isolated using a previously published protocol (Ju et al. 2003). In brief, TRIzol reagent (Invitrogen, CA) was used to isolate total RNA from snap frozen tissues. RNA was treated with DNase I (Promega, WI). The integrity of total RNA was determined by 1% formaldehyde-agarose gel electrophoresis. cDNA synthesis was carried out with the Superscript III cDNA synthesis kit (Invitrogen, CA) using 1 μg of total RNA as the template and specific reverse primers (Supplementary Table) under 65°C, 5 min, and 50°C, 60 min of reverse transcription. A total of three fragments were synthesized for further analysis.

### Mutation Detection of p53 by PCR and Sequencing

The PCR reaction was carried out in a 25 μl reaction mixture containing cDNA 2 μl, 10x PCR golden buffer 2.5 μl, 1.5 mM MgCl2, 200 μM dNTP (Ambion, TX), 5 pmol primers and 1.25 U of AmpliTAq Gold polymerase plus 1M betaine (Sigma, MI). All PCR reagents were from Applied Biosystems Inc. except where mentioned. The reaction was initiated at 95°C for 10 min. Thermal cycling was as follows: denaturation at 95°C for 30 sec, annealing using touchdown from 62°C to 55°C for 30 sec (0.5°C decrement each round), extension at 72°C for 35 sec followed by an additional 25 rounds of 95°C 30 sec, 55°C 30 sec and 72°C 40 sec. Final extension was carried out at 72°C for 10 min on the PTC-225 Peltier Thermal Cycler (Bio-Rad, MA). PCR products were purified using a MultiScreen-PCR purifying plate (Millipore, MA) and submitted for sequencing. The PCR and sequencing primers were designed with Primer Premier (version 5.0) software (PREMIER Biosoft International, CA) and are listed in the Supplementary Table. Sequencing was performed with the BigDye Terminator v3.1 Cycle Sequencing Kit from Applied Biosystems, Inc. (CA). Five microliters (50 ng) of template DNA was added to wells of a 96-well plate containing 15 μl of sequencing cocktail consisting of 0.4 μl premix from sequencing kit, 7.6 μl 2.5 × sequencing buffer, 0.5 μl 10 mM primer and 6.5 μl water. Sequencing reactions were carried out for 35 cycles (96°C, 10 sec; 50°C, 5 sec; 60°C, 2 min 30 sec). The products were precipitated with 50 μl 100% ethanol and 2 μl 3M NaOAc (pH 4.8), and pellets were rinsed with 70% ethanol. After addition of 10 μl Hi-Di Formamide (Applied Biosystems Inc, CA) and denaturing at 94°C for 10 min, samples were loaded onto an ABI 3730xl sequencer. Sequences were analyzed with SeqManII from DNASTAR, Inc. by comparing with *Homo sapiens* tumor protein p53 mRNA sequence (NM_000546). All allelic variations were recorded.

### miRNA Reverse Transcription and qRT-PCR Analysis

mirVana^™^ qRT-PCR Primer Sets (Ambion Inc. TX) for miRNA specific reverse transcription including hsa-miR-30a-5p, hsa-miR-181b, hsa-let-7g, hsa-miR-26a, hsa-let-7b, has-miR-15b, has-miR-27a, has-miR-200c, has-miR-191, has-miR-30c, and endogenous control 5S rRNA were utilized according to the manufacture’s protocol. Briefly, the reaction master mix containing mirVana^™^ 5 × RT Buffer, 1 × mirVana^™^ RT Primer, Array-Script^™^ Enzyme Mix and nuclease-free water was mixed with 20 ng of each total RNA sample. These mixtures were incubated for 30 min at 37°C, then 10 min at 95°C. qRT-PCR was carried out using the Applied Biosystems 7500 Real-Time PCR System (Applied Biosystems Inc. CA) and mirVana^™^ qRT-PCR miRNA Detection Kit (Ambion Inc. TX). The PCR master mix containing *mir*Vana^™^ 5 × PCR Buffer (with SYBR® Green I), 50 × ROX, SuperTaq^™^ Polymerase, *mir*Vana^™^ PCR Primers, and RT products was processed as follows: 95°C for 3 min, and then 95°C for 15 sec, 60°C for 35 sec for up to 40 cycles (n = 3). Signal was collected at the endpoint of every cycle.

### Statistical Analysis

The gene expression ΔCT values of miRNAs from each sample were calculated by normalizing with internal control 5S rRNA and relative quantitation values were plotted using SDS software v1.2 (Applied Biosystems Inc. CA). The statistical studies were performed using MedCalc® for Windows, version 7.4.2.0 (MedCalc software, Belgium). The statistically significant differences in expression level between tumor and normal tissues for each target were calculated using a paired Wilcoxon test. The Logrank test for the generated Kaplan-Meier curve was conducted to evaluate the association between the expression level of each miRNA and survival rate. The cut-off was set to p <0.05.

## Results

### Overexpression of miRNAs in Colorectal Cancer

**Figure 1:**
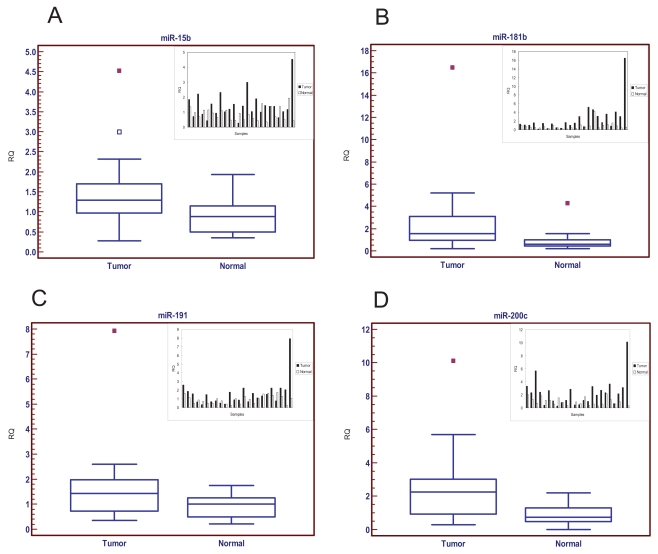
miRNA expression in colorectal cancer and normal tissue specimens. Gene expression values were expressed as ratios between miRNAs with an internal control 5S ribosomal gene. (A) *hsa-miR-15b* expression (p = 0.0278). (B) *hsa-miR-181b* expression (p = 0.0002). (C) *hsa-miR-191* expression (p = 0.0264). (D) *hsa-miR-200c* expression (p = 0.0017). The statistical significance was evaluated by a two tailed paired Wilcoxon test. The small charts in the upper right corners display the miRNA expression for all individual paired samples (24 Tumors vs. Normal Samples).

**Figure 2:**
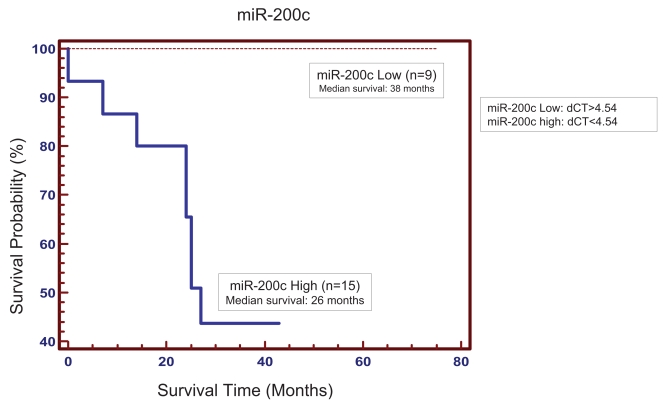
Kaplan-Meier overall survival curve based on *hsa-miR-200c* expression (p = 0.0122, Logrank test).

In this study, the expression levels of 10 different miRNAs known to be deregulated based on our previous studies were quantified using miRNA specific qRT-PCR analysis. Among these, four miRNAs were found to be overexpressed in colorectal cancer samples. The expression of *hsa-miR-15b* was overexpressed by nearly 1.5-fold (Median: 1.30 vs. 0.89, p = 0.0278) (Figure 1A). The expression of *hsa-miR-181b* was elevated 2.5-fold (Median: 1.54 vs. 0.61, p = 0.0002) in tumor samples (Figure 2). The expression of *hsa-miR-191* was significantly enhanced 1.4-fold (Median: 1.44 vs. 1.01, p = 0.0264) in colorectal tumors (Figure 1C). The expression of *hsa-miR-200c* was also significantly upregulated 3-fold in tumor specimens (Median: 2.25 vs. 0.75, p = 0.0017) (Figure 1D).

### Evaluation of Prognostic Values of miRNAs

To further evaluate the clinical relevance of these overexpressed miRNAs in colorectal cancer in terms of prognosis, Kaplan-Meier survival analysis was performed using patient overall survival. Our results indicated that *hsa-miR-200c* was significantly associated with patient survival (Figure 2). Patients (n = 9) with low expression of *hsa-miR-200c* (ΔCT less than 4.54) tended to have longer survival (median survival of 38 months vs. 26 months) than patients (n = 15) with higher levels of *hsa-miR-200c* expression (p = 0.00122). The expression of *hsa-miR-200c* was not related to the difference in tumor stage (data not show).

### Correlation of p53 Status and miRNA expression

**Figure 3:**
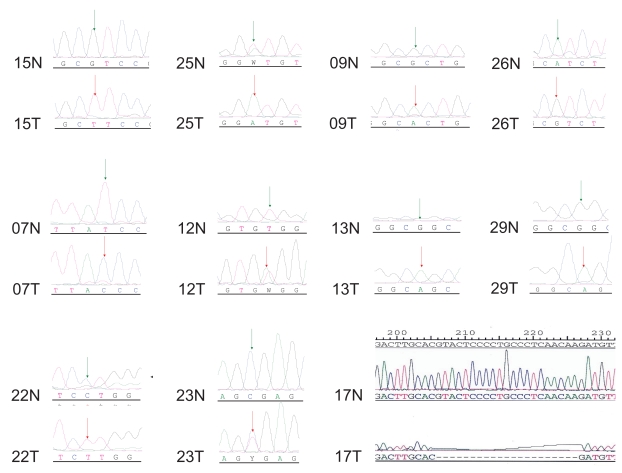
Sequence analysis of p53 cDNA. A total of 11 samples have p53 mutations/deletion in the coding region. Numbers refer to individual patients. N = normal; T = tumour tissue.

**Figure 4:**
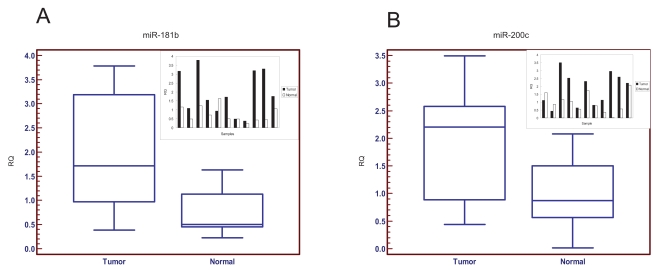
Correlation of p53 status and the expression of miRNAs. (A) *hsa-miR-181b* expression (p = 0.0098). (B) *hsa-miR-200c* expression (p = 0.0322). The statistical significance was evaluated by a two tailed paired Wilcoxon test. The small charts in the right corner show the miRNA expression of all individual paired samples with p53 mutations/deletion (11 Tumors vs. Normal Samples).

Our previous studies showed that over 46% of the putative promoter sites of 324 miRNAs contain p53 binding site(s). We thus decided to sequence the p53 gene’s coding sequence in all 48 patient samples. It turned out that over 46% of colorectal tumors contained p53 mutations or deletions (Table 2). Representative p53 sequence traces around the mutation and deletion regions are illustrated in Figure 3. The expression of *hsa-miR-181b* was 3.4-fold higher in 11 tumors with p53 mutations/deletions than the corresponding normal samples with wild type p53 and was strongly associated with p53 mutation status (median: 1.72 vs. 0.50, p = 0.0098) (Figure 4A). The expression of *hsa-miR-200c* was 2.5-fold higher in the 11 tumors containing p53 deletions/mutations than the corresponding counterparts and was also strongly associated with the p53 mutation status (median: 2.20 vs. 0.87, p = 0.03) (Figure 4B). We also compared the expression status of miRNAs within the tumors with or without p53 mutations. The results also showed a significant correlation of *hsa-miR-200c* and *hsa-miR-181b* with p53 mutation status (data not show). Both of these miRNAs contain p53 binding site(s) in the putative promoter regions.

## Discussion

Many recent efforts in the field of cancer research have focused on miRNA biology. Even a small change in miRNA expression can cause a profound effect on the gene expression of hundreds of mRNAs at the post-transcriptional or translational level. Mounting evidence has shown that miRNAs are involved in cancers such as lymphoblastic leukemia, glioblastoma, B-cell chronic lymphocytic leukemia (B-CLL), and many solid tumors including colon cancer (Benard and Douc-Rasy, 2005; Calin et al. 2004; Cimmino et al. 2005; Cummins et al. 2006).

Previous studies from our group have identified many deregulated miRNAs associated with the p53 tumor suppressor gene (Xi et al. 2006). As a transcription factor, p53 regulates its downstream genes to mediate cell cycle control and apoptosis (Bunz et al. 1998; Bunz et al. 1999; Wang et al. 2001; Yu et al. 2002). We provided evidence that p53 may also act as a tumor suppressor by regulating certain miRNAs (Xi et al. 2006). p53 is one of the most frequent mutated tumor suppressor genes in colorectal and other cancer types (Hollstein et al. 1991). In this study, we further investigated the clinical relevance of some of the miRNAs overexpressed due to the loss of p53 in colorectal cancer. These overexpressed miRNAs may be involved in the oncogenic process.

We chose 24 paired normal colon tissues and cancer specimens for the expression analysis of 10 miRNAs. This is a valid set of clinical samples with which to evaluate the clinical relevance of miRNAs in colorectal cancer. The sample size is small, but unlike mRNA marker discovery starting from over 40,000 genes, which may need at least 100 samples to obtain reliable markers, there are only a few hundred known miRNAs in mammals. Therefore, we are working with up to 100-fold less marker candidates compared to mRNA transcript profiling. This is in fact a major advantage of using miRNAs as potential biomarkers instead of mRNAs. Another advantage is that each miRNA can potentially regulate several hundred genes at the post-transcriptional level. Profiling these master regulators may be superior to profiling their downstream mRNA targets. Four of the ten miRNAs were overexpressed in colorectal cancer samples (Figure 1). These results have been further confirmed in another study by our group (manuscript under review) and others (Cummins et al. 2006). Each of these miRNAs is capable of potentially regulating well over 100 target mRNAs via imperfect base pairing. Some of these miRNAs have recently been reported to function as potential oncogenes (Hammond, 2006). The *hsa-miR-200c* is strongly associated with overall patient survival (Figure 2). *hsa-miR-200c* can potentially regulate over 200 target genes including *MAPKKK3, eIF-4E, RAS homologs, RNA polymerase II, and cyclin L1* based on bioinformatics analysis (John et al. 2004). Thus, in some way, miRNAs can be viewed as master regulators. This study may provide some candidate miRNAs as potential targets for new therapeutics development. The detailed molecular and cellular mechanisms underlining the action of these miRNAs will be investigated in our future studies.

The p53 status in these samples was determined via sequencing of the p53 cDNAs (Figure 3 and Table 2). Nearly 50% of the tumors contained p53 mutations/deletions and this was highly consistent with many previous reports that p53 is one of the most frequently mutated tumor suppressor genes in cancer (Tomita et al. 1999). Among the tumors with mutated p53, both *hsa-miR-181b* (Figure 4A) and *hsa-miR-200c* (Figure 4B) were highly over-expressed compared to the paired normal samples. This is consistent with our previous report that p53 may potentially mediate miRNA expression to exert its tumor suppressor function. The loss of p53 tumor suppressor function will activate some of these potential oncogenic miRNAs. Many genes such as *cytochrome C, ECIP-1, MAPPKKK1, TEM6, E2F5, GATA6, PP2B,* and eIF5A, are predicted to be regulated by *hsa-miR-181b.* These genes have been shown to be important for cell signaling, cell cycle control and chemosensitivity. We speculate that the miRNAs can modulate expression of a number of genes at the translational level.

In conclusion, in this study, the *in vivo* significance of 10 mature miRNAs was evaluated in 24 matched normal and colorectal cancer patient samples. The expression of *hsa-miR-15b, hsa-miR-181b, hsa-miR-191* and *hsa-miR-200c* were significantly over-expressed in colorectal cancer patients and they may be associated with the development of the disease. The expression of *hsa-miR-200c* was strongly associated with overall patient survival. That expression of *hsa-miR-200c* was not related to the different stages of the disease further supports this notion. We believe that this is just the beginning of trying to understand how miRNAs (the micrornanome) are involved in colorectal and perhaps other cancers. Large follow up studies will be necessary in the near future to further confirm the significance of these miRNA markers. Modulation of miRNAs will likely be a new frontier for cancer diagnosis and treatment (Hampton, 2005).

## Supplementary Material

**Supplementary Table:** Primer sets used for RT, PCR amplification and sequencing of p53 gene.

Fragments	Primers	Lengths	Range
1	F^a^: AAGTCTAGAGCCACCGTCCA R^a^: TCTGGGAAGGGACAGAAGA	439	From+115 to +553
2	F: GAAGACCCAGGTCCAGATGA R: AGCTGTTCCGTCCCAGTAGA	643	From+417 to +1059
3	F: CCATCCTCACCATCATCACA R: GCAAGCAAGGGTTCAAAGAC	528	From+1000 to +1527

a F and R refer to forward and reverse primers, respectively.

Both forward and reverse primers were used for sequencing. Reverse primer was used for reverse transcription reaction.

## Figures and Tables

**Table 1:** Clinical features of the 24 patients in the series used in this study.

Characteristics	Frequency	Percentage (%)
**Age(Years)**		
Mean(range)	62(30–93)	
**Gender**		
Male	14	58.3
Female	10	41.7
**Anatomic site**		
Ascending colon	3	12.5
Transverse colon	2	8.3
Descending colon	4	16.7
Sigmoid colon	3	12.5
Rectum	12	50.0
**Histology**		
Adenocarcinoma	24	100
**UICC stage**		
I	4	16.7
II	4	16.7
III	8	33.3
IV	8	33.3
**Survival (Months)**		
Mean(range)	30(0–75)	
0–20	3	12.5
20–50	20	83.3
>50	1	4.2

**Table 2:** p53 mutations confirmed by cDNA sequencing in all samples.

#	Sample	cDNA-position	Normal	Tumor	Normal	Tumor
1	15	720	GTC	TTC	Leu	Ser
2	25	766	G TT/G AT	G AT	Val/Asp	Asp
3	09	775	C GC	C AG	Arg	Gln
4	26	829	C AT	C GT	His	Arg
5	07	835	A TC	A CC	Ile	Thr
6	12	859	G TG	G TG/G AG	Val	Val/Glu
7	13	984	GGC	AGC	Gly	Ser
8	29	984	GGC	AGC	Gly	Ser
9	22	1084	C CT	C TT	Pro	Leu
10	23	1167	CGA	CGA/ TGA	Arg	Arg/Ter(Stop)
11	17	626–646	Deletion			
